# Intraguild predation by the predatory soil mite *Blattisocius mali* (Acari: Blattisociidae) and its predation preference towards conspecific and heterospecific prey

**DOI:** 10.1007/s10493-026-01198-9

**Published:** 2026-10-03

**Authors:** Manoj Kumar Jena, Marcin Studnicki, Katarzyna Michalska

**Affiliations:** 1 Department of Plant Protection, Institute of Horticultural Sciences, Warsaw University of Life Sciences, Nowoursynowska 159, Warsaw, 02-776 Poland; 2 Department of Biometry, Institute of Agriculture, Warsaw University of Life Sciences, Nowoursynowska 159, Warsaw, 02-776 Poland

**Keywords:** Biological control, Competition, Edaphic predatory mite, Generalist, Predator-predator interaction

## Abstract

The predatory soil mites *Blattisocius mali* and *Stratiolaelaps scimitus* share similar prey and occupy similar habitats. This study explores intraguild predation (IGP) by various life stages of *B. mali* on the developmental stages of *S. scimitus* across different starvation periods, and their predation preference for conspecific and heterospecific eggs or larvae in the absence of shared prey at 25 °C, 85 ± 5% RH, and a 16 L:8D photoperiod. All feeding developmental stages of *B. mali* consumed *S. scimitus* eggs and larvae; however, they did not kill *S. scimitus* deutonymphs or later developmental stages. Few *B. mali* females killed *S. scimitus* protonymphs, whereas other developmental stages of *B. mali* did not kill these protonymphs. Interestingly, the propensity of *B. mali* females to eat heterospecific eggs was not affected by the starvation period. However, their predation on heterospecific larvae increased with increasing starvation period up to 48 h and then stabilized. *Blattisocius mali* females, males, or deutonymphs showed a greater propensity towards heterospecific larvae compared to eggs across all the tested starvation periods. Notably, the proportion of *B. mali* protonymphs that ate heterospecific eggs was significantly higher than that of other stages, and males had a significantly greater tendency to prey on heterospecific larvae relative to other stages. When given a choice, all life stages of *B. mali* clearly distinguished between conspecific and heterospecific eggs, preferring to prey on conspecific eggs. In contrast, when selecting between conspecific and heterospecific larvae, they preferred to prey upon heterospecific larvae. This study suggests that *B. mali* may interact with *S. scimitus* and feed on their eggs and larvae. Further studies are necessary to investigate the effect of IGP between *B. mali* and *S. scimitus* on their survival, reproduction, population dynamics, and ultimately pest suppression.

## Introduction

Interactions both within and between predator species play a crucial role in shaping predator-prey and predator-predator relationships. Understanding these interactions is essential for predicting how predators influence the natural and biological control of arthropod pests (Polis et al. 1989; Sih et al. 1998; Loosey and Denno 1999; Tsuchida et al. 2022). Two common forms of trophic interactions among predators are intraspecific interactions, in which individuals of the same species kill and consume one another, and interspecific interactions, in which different predator species kill and consume heterospecific rivals (Polis et al. 1989; Elgar and Crespi 1992; Yadav and Omkar 2026). Both intraspecific and interspecific interactions are widespread across the animal kingdom (Polis et al. 1989; Elgar and Crespi 1992; Chi et al. 2026b; Gong et al. 2026). These interactions tend to intensify when prey is scarce, as they can provide additional food sources for predators (Polis et al. 1989; Schausberger 1997; Cai et al. 2026). Such interactions can significantly influence the distribution and population dynamics of the species involved. They often lead to spatial and temporal segregation, causing prey species to be displaced or confined to less optimal habitats or periods, which can affect their survival and abundance (Hurd and Eisenberg 1990; Moran et al. 1996; Walzer et al. 2001).

Interspecific interactions can arise among predators within (intraguild) or outside (extraguild) their immediate ecological guilds. Intraguild predation (IGP) specifically occurs within biological control systems when one natural enemy preys upon another species of natural enemy while simultaneously competing for the same prey (Janssen et al. 2006). Beyond providing direct nutritional benefits, IGP can also diminish competition for shared prey and reduce the risk of predation on oneself or related species (Polis et al. 1989). This phenomenon can significantly impact ecological communities, influencing species diversity, population dynamics, ecosystem stability, and the structure of food webs (Schausberger and Croft 2000a; Döker et al. 2021). Factors such as prey availability, predator density, and the frequency of predator encounters can promote IGP. Additionally, traits such as body size, feeding behaviour, dietary preference, life history strategy, and habitat complexity play crucial roles in shaping the intensity and direction of IGP (Polis et al. 1989; Shakya et al. 2009; Mendel and Schausberger 2011). Generally, generalist predators tend to be more aggressive toward other predators than specialists (Schausberger and Croft 2000a), and they often gain equivalent or greater nutritional benefits from IGP (Schausberger and Croft 2000b). IGP is widespread in both terrestrial and aquatic ecosystems among generalist predators (Polis et al. 1989; Polis and Holt 1992; Chi et al. 2026b; Liu et al. 2026). In biological control practices, different agents are often deployed together (Yano 2005). Sometimes, two or more predator species with complementary effects are released simultaneously. For example, Yano (2005) noted that an initial release of an easily reared predator is used as a preventative measure against pests. Once pest populations reach high levels, a second predator that may be more challenging to mass-rear is introduced to further suppress the pest population.

Soil-dwelling mites, such as *Blattisocius mali* (Oudemans) (Acari: Blattisociidae) and *Stratiolaelaps scimitus* (Womersley) (Acari: Laelapidae), are generalist predators. Both species can prey upon various arthropod pests, including the mould mite *Tyrophagus putrescentiae* (Schrank) (Acari: Acaridae), fungus gnat *Lycoriella* sp. (Diptera: Sciaridae), western flower thrips *Frankliniella occidentalis* (Pergande) (Thysanoptera: Thripidae), and nematodes (Brødsgaard et al. 1996; Enkegaard et al. 1997; Steiner et al. 1999; Navarro-Campos et al. 2016; Abbas et al. 2020; Asgari et al. 2023; Michalska et al. 2023). Studies have shown that *B. mali* is particularly effective at suppressing *T. putrescentiae* populations, demonstrating strong potential to control mite infestations in stored flower bulbs and young cucumber plants (Jena et al. 2024, 2025a; 2025b, 2026a, 2026b, 2026d; Michalska et al. 2025). Similarly, *S. scimitus* has proven to be highly effective against thrips infesting greenhouse crops and sciarid flies in horticultural and mushroom systems (Castilho et al. 2009; Lan et al. 2025; Chi et al. 2026a). When *B. mali* is released into a crop, it may interact with other predatory mites, including *S. scimitus*, which are present both in natural environments, such as soil or litter, and also used in the biological control of crop pests (McMurtry and Croft 1997; McMurtry et al. 2013; Moreira and de Moraes 2015). These interactions can lead to competition or even predation among different predatory mite species, which might undermine the effectiveness of biological control efforts aimed at managing pests.

This study aimed to investigate the intraguild predation by *B. mali* on *S. scimitus* immatures and its predation preference towards conspecific and heterospecific prey. Recognising that a predator’s hunger level can influence its predatory behaviour (Zhang and Zhang 2022; Jena et al. 2026c), we expect that (1) prolonged starvation may increase *B. mali’s* tendency to target heterospecific prey. Given that *B. mali* shows a higher predation rate on its own larvae than on its eggs (Jena et al. 2026c), we predict that (2) the degree of intraguild predation may be greater on larval stages than on eggs. Considering that female *B. mali* exhibits greater voracity than males (Jena et al. 2025a), we expect that (3) *B. mali* females are more likely to prey upon heterospecifics than males. Acknowledging the variation in predation rates across different life stages of *B. mali* towards prey (Jena et al. 2025a), we expect that (4) *B. mali* deutonymphs may engage more frequently in intraguild predation than other developmental stages. Finally, based on previous findings that adult females of generalist predators can distinguish between conspecific and heterospecific prey and tend to prefer heterospecifics when given a choice (Schausberger 1999; Walzer and Schausberger 1999; Meszaros et al. 2007; Momen and Abdel Khalek 2009), we hypothesise that (5) *B. mali* may also show a preference for preying on heterospecifics when both conspecific and heterospecific prey are available.

## Materials and methods

### Predator and prey sources and rearing: general methods

The primary colony of the predatory mite *B. mali* was taken from the Department of Plant Protection of Warsaw University of Life Sciences (WULS), Warsaw, Poland. The colony was kept on water-soaked foam pads using wheat bran as the growing medium, within the same department at WULS (Jena et al. 2024, 2025a; 2025b). Every week, the mites were provided with a blend of various life stages of *T. putrescentiae* as food. The mites were housed in a climate-controlled room maintained at 23 ± 1 °C, 85 ± 5% RH, and a 16 L:8D photoperiod.

The main colony of the predatory mite *S. scimitus*, provided as the Entomite-M formulation, was brought from Koppert Polska. This colony was maintained in plastic containers measuring 120 mm in diameter and 200 mm in height, using damp peat as a growing medium. The containers were sealed with lids featuring small vents for air circulation and kept in a room at 23 ± 1 °C, 95 ± 5% RH, and in complete darkness at the Department of Plant Protection at WULS. The acidic sphagnum peat (KIK Ziemskie Produkty Sp. z o.o., Poland) had a pH level between 3.5 and 4.5, contained over 85% organic matter, had a salinity of less than 1.0 g/l, and consisted of particles ranging from 0 to 20 mm. Before use, the peat was sterilized in a Memmert steriliser at 100 °C for 5 h and subsequently cooled with a fine mist of distilled water. The colony was provided with a weekly diet consisting of various life stages of *T. putrescentiae*. Efforts were made to ensure the colony remained moist and protected from contamination by harmful fungi.

To obtain an adequate number of eggs, the *T. putrescentiae* culture was sourced from and maintained at the mass-rearing facility of the Department of Plant Protection at WULS. This culture was maintained in glass Petri dishes using equal amounts of instant dry baker’s yeast and wheat bran, and kept at 26 °C and 95 ± 5% RH. The setup was kept in total darkness inside a Panasonic Sanyo Environmental Test Chamber (Jena et al. 2024).

To obtain eggs, larvae, protonymphs, and deutonymphs of *B. mali* for experiments, gravid females were randomly selected from the colony. Each *B. mali* female was placed individually in a Plexiglass cage chamber containing a mix of life stages of *T. putrescentiae* as food for 20 h before starting the experiments. The Plexiglass cage featured a round opening (8 mm), as detailed by Jena et al. (2024). These mite-containing cages were kept inside a desiccator at 25 °C, 85 ± 5% RH, and a 16 L:8D photoperiod, all maintained in a Panasonic-cooled incubator (MIR-154-PE, Japan). After 20 h, the eggs produced were collected and reared individually with various life stages of *T. putrescentiae* as food to obtain larvae, along with well-fed protonymphs and deutonymphs. The newly hatched larvae were raised individually in cages without food to obtain unfed protonymphs. Older protonymphs, just before they were about to moult, were taken and kept without food to obtain unfed deutonymphs. To obtain well-fed females and males, *B. mali* individuals were randomly selected from the colony and kept with mixed life stages of *T. putrescentiae* as prey for 24 h. After this period, these well-fed females and males were starved for designated periods (24, 48, 72, or 96 h) to obtain starved predators.

To procure eggs, larvae, and protonymphs of *S. scimitus* for experiments, gravid *S. scimitus* females were randomly selected from the stock colony and placed individually in the chamber of a Plexiglass cage with *T. putrescentiae* mixed life stages as prey for 20 h before commencing the trials. *Stratiolaelaps scimitus* eggs and larvae are sensitive to dry conditions. To retain moisture, Scotch tape was attached to tissue paper at the bottom of the cage. The cages with mites were kept in a desiccator at 25 °C, 85 ± 5% RH, and a 16 L:8D photoperiod in an incubator. The 20 h eggs obtained were collected and reared individually with *T. putrescentiae* mixed life stages as prey to obtain larvae, well-fed protonymphs, and well-fed deutonymphs. To obtain well-fed females and males, *S. scimitus* individuals were randomly selected from the colony and kept with mixed life stages of *T. putrescentiae* as prey for 24 h.

### Experimental procedure

The intraguild predation and preference experiments were performed using a Plexiglass cage, in which a conical-shaped hole was drilled instead of a round hole (8 mm) (Jena et al. 2024, 2026a; Ibrahim et al. 2026), and Scotch tape was attached along with tissue paper at the bottom of the cage to keep it moist. The experimental cages were placed in a desiccator in an incubator maintained at 25 °C, 85 ± 5% RH, and a 16 L/8D photoperiod.

### Intraguild predation of *Blattisocius mali* in the absence of alternative prey

All feeding developmental stages of *B. mali*, i.e., protonymph, deutonymph, adult female, or adult male, were used as predators, while the 20 h egg, 2 h larva, 4 h protonymph, 4 h deutonymph, and well-fed adult female and male of *S. scimitus* were used as heterospecific prey candidates. The effects of predator starvation, prey type, and predator type were examined (Table 1). One well-fed *B. mali* protonymph, deutonymph, adult female, or adult male was tested in combination with one well-fed *S. scimitus* protonymph, deutonymph, adult female, or adult male. Each well-fed or unfed *B. mali* protonymph or deutonymph was combined with either one 20 h egg or a 2 h larva of *S. scimitus* as prey. Each *B. mali* adult male, starved for 0, 24, or 48 h, was taken with either one 20 h egg or a 2 h larva of *S. scimitus*. Each *B. mali* adult female, starved for 0, 24, 48, 72, or 96 h, was tested with either one 20 h egg or a 2 h larva of *S. scimitus*. Each predator-prey combination was replicated 30 times. Twenty hours after the start of the experiment, whether the heterospecific prey was preyed upon by predators was recorded.

**Table 1:** Predator-prey combinations used in the intraguild predation experiments of *Blattisocius mali* in the absence of alternative prey

Predatory mite (Blattisocius mali)	Heterospecific prey (Stratiolaelaps scimitus)	Number of replications (*n*)
Well-fed protonymph	Well-fed protonymph, deutonymph, adult female, or adult male	30
Well-fed deutonymph	Well-fed protonymph, deutonymph, adult female, or adult male	30
Well-fed female	Well-fed protonymph, deutonymph, adult female, or adult male	30
Well-fed male	Well-fed protonymph, deutonymph, adult female, or adult male	30
0, 24, 48, 72, or 96 h- starved female	20 h egg	30
	2 h larva	30
0, 24, or 48 h starved male	20 h egg	30
	2 h larva	30
Well-fed or unfed protonymph	20 h egg	30
	2 h larva	30
Well-fed or unfed deutonymph	20 h egg	30
	2 h larva	30

### Preference of *Blattisocius mali* to conspecific and heterospecific prey

All developmental stages of *B. mali* that ingest food (unfed protonymph, unfed deutonymph, 72 h starved adult female, or 24 h starved male) were used as predators, and the 20 h egg or 2 h larva of both *B. mali* (conspecifics) and *S. scimitus* (heterospecifics) were used as prey candidates. Since preliminary observations showed that most of the 24 h- or 48 h-starved *B. mali* females did not choose either conspecific or heterospecific prey during the experimental period (12–24 h), 72 h-starved females were used. Each predator was provided with 3 eggs or larvae of both conspecifics and heterospecifics. The eggs and larvae of *S. scimitus* were distinguished from those of *B. mali* by size; the eggs and larvae of *S. scimitus* are much larger than those of *B. mali*. Twenty-four hours after the start of the experiment, the number of conspecific and heterospecific eggs preyed upon by the predator was recorded. Twelve hours after the start of the experiment, the number of larvae preyed upon was noted due to the shorter developmental period of larvae. Each predator-prey combination was replicated 20 times.

### Statistical analysis

The proportion of predators that consumed heterospecific prey among all tested predators of each type was analysed using a Generalised Linear Model (GLM) with a binomial distribution and a logit link function. In contrast, prey preference among different life stages of *B. mali* was assessed by comparing the proportions of conspecific and heterospecific prey consumed using a GLM with a Poisson distribution and a log link function. In both analyses, post hoc pairwise comparisons were performed using Tukey’s adjustment for multiple comparisons. All statistical analyses were conducted using R software (R Core Team 2025).

## Results

### Intraguild predation of *Blattisocius mali* in the absence of alternative prey

All feeding developmental stages of *B. mali* ate eggs and larvae of *S. scimitus*. Only a very low proportion of *B. mali* females, specifically four out of thirty, were observed to kill *S. scimitus* protonymphs. The remaining twenty-six *B. mali* females were killed by *S. scimitus* protonymphs. Conversely, all *B. mali* protonymphs, deutonymphs, and adult males were killed and eaten by *S. scimitus* protonymphs and more advanced developmental stages.

**Fig. 1:**
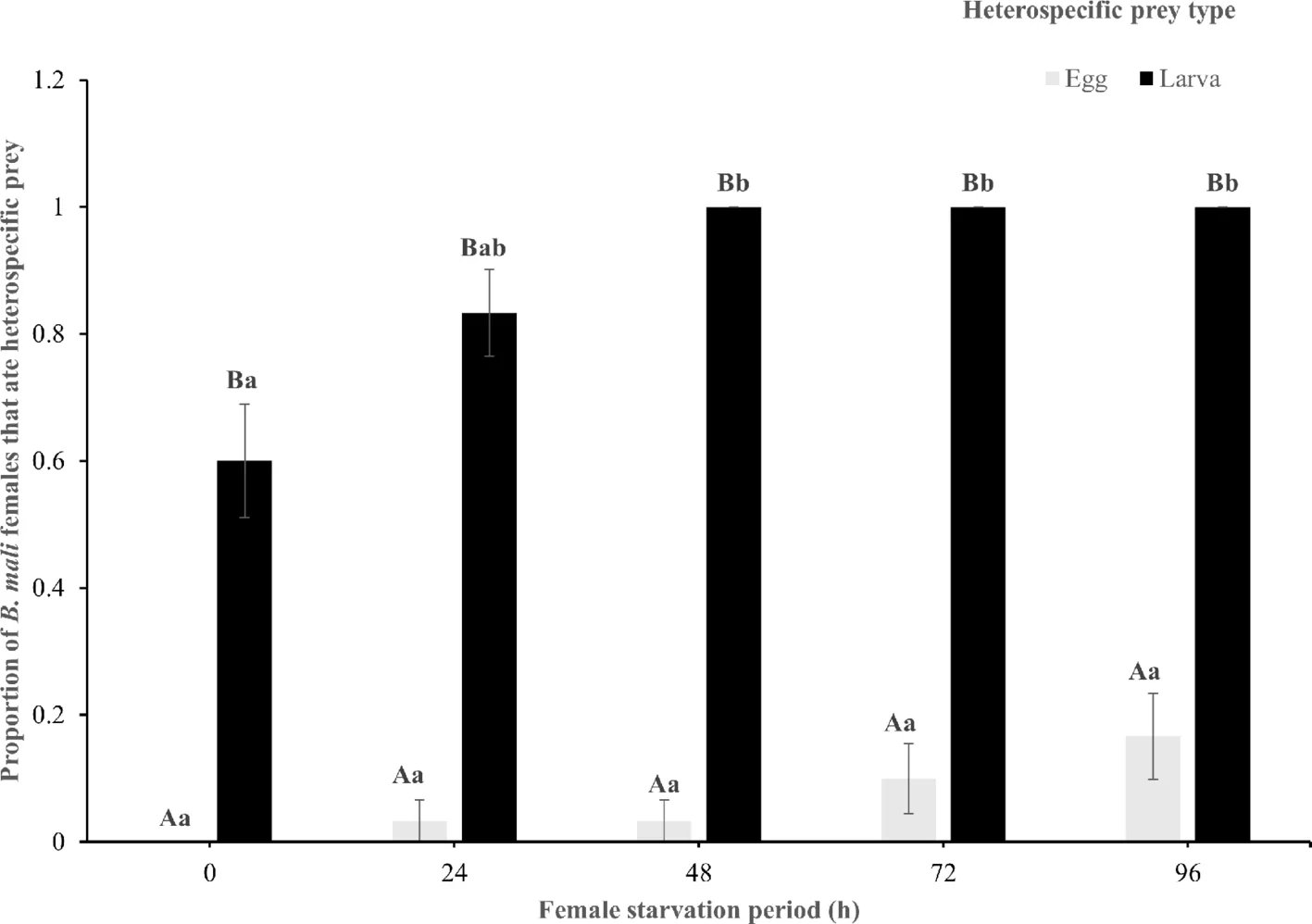
Mean (*± SE*) proportion of *Blattisocius mali* adult females (*n = 30), of the tested starvation periods, that ate heterospecific prey. Different uppercase letters indicate significant differences between prey types at each starvation period, while lowercase letters indicate significant differences among starvation periods of *B. mali* preying on the same prey (*P* < 0.05; Tukey test)

When examining the behaviour of *B. mali* females towards heterospecific prey, it was found that the mean proportion of *B. mali* females (out of all tested individuals) that ate heterospecific prey was significantly affected by the starvation period (*χ²* = 12.6, df = 4, *P* < 0.05) and prey type (*χ²* = 266.9, df = 1, *P* < 0.0001). However, there was no significant interaction between starvation period and prey type (*χ²* = 4.1, df = 4, *P* = 0.39). Specifically, the proportion of well-fed (i.e., 0 h starved) *B. mali* females that ate heterospecific larvae was similar to that of 24 h starved females (*P* > 0.05), but it was significantly lower than that of *B. mali* females starved for 48 h or longer (*P* < 0.05) (Fig. 1). Conversely, the proportion of *B. mali* adult females that ate heterospecific eggs was similar among starvation periods (*P* > 0.05). Additionally, the proportion of *B. mali* females that ate heterospecific larvae was significantly higher than that of those females that ate heterospecific eggs (*P* < 0.05) (Fig. 1).

**Fig. 2:**
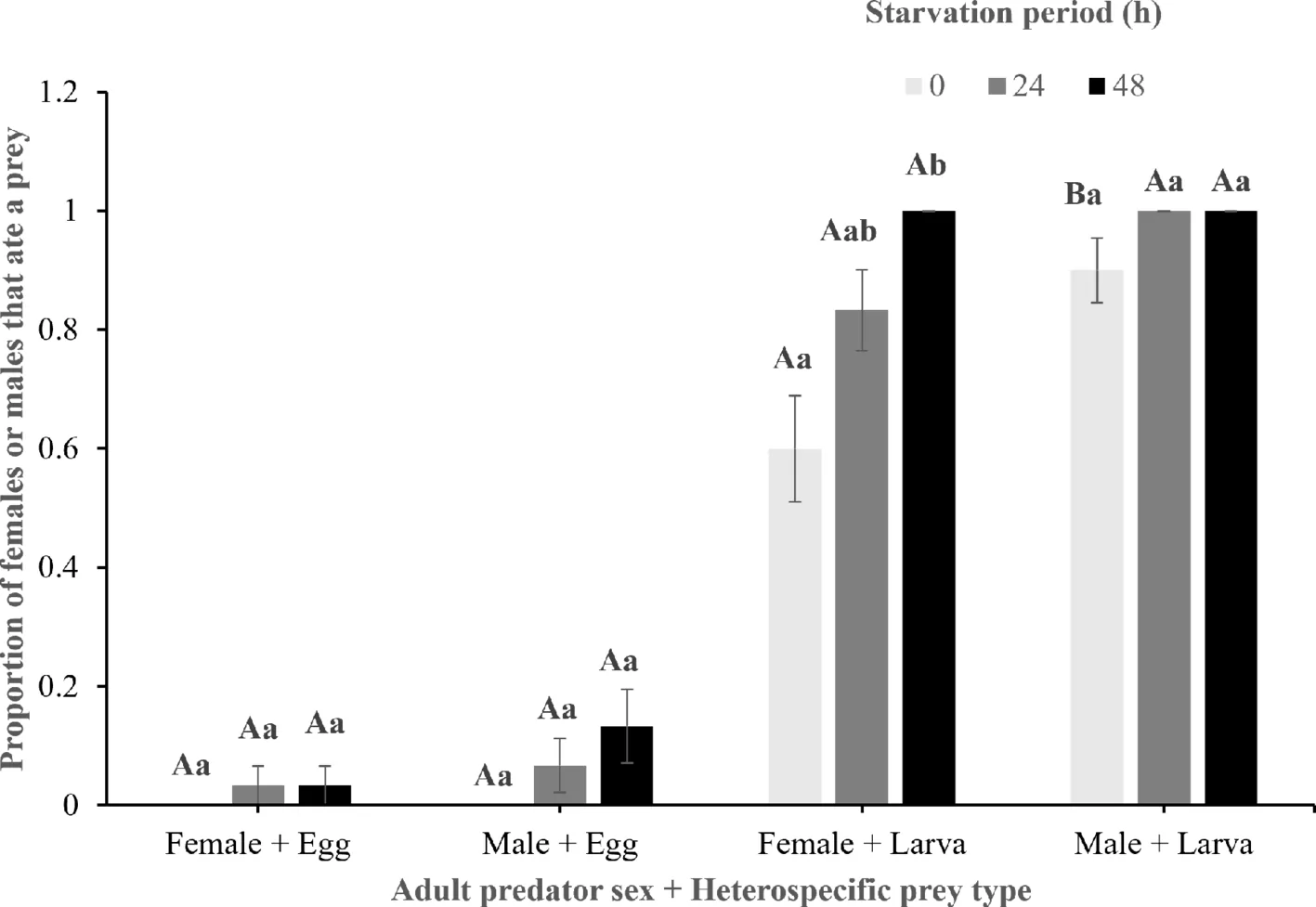
Mean (*± SE*) proportion of *Blattisocius mali* adult females or males (*n = 30), of various starvation periods, that ate heterospecific prey. Different uppercase letters indicate significant differences between female and male *B. mali* that ate the same prey, while lowercase letters indicate significant differences among starvation periods of female or male *B. mali* preying on the same prey (*P* < 0.05; Tukey test)

When assessing the behaviour of *B. mali* females or males towards heterospecific prey, it was observed that the proportion of *B. mali* consuming heterospecific eggs was significantly affected by the starvation period (*χ²* = 7.2, df = 2, *P* < 0.05). However, neither the predator sex (*χ²* = 2.2, df = 1, *P* = 0.13) nor the interaction between the starvation period and predator sex (*χ²* = 0.2, df = 2, *P* = 0.90) showed a significant effect. In contrast, the proportion of *B. mali* individuals consuming heterospecific larvae was significantly affected by both the starvation period (*χ²* = 23.7, df = 2, *P* < 0.0001) and predator sex (*χ²* = 13.5, df = 1, *P* < 0.001). Yet, the interaction between these two factors remained non-significant (*χ²* = 1.5, df = 2, *P* = 0.47). The proportion of 0 h, 24 h, or 48 h starved *B. mali* males that ate heterospecific eggs or larvae was similar (*P* > 0.05) (Fig. 2). The proportion of *B. mali* males that ate heterospecific eggs was similar to that of females consuming the same prey (*P* > 0.05). Interestingly, the proportion of well-fed *B. mali* males that ate heterospecific larvae was significantly higher than that of well-fed females consuming the same prey (*P* < 0.05). Conversely, the proportion of 24–48 h starved *B. mali* males that ate heterospecific eggs was similar to that of 24–48 h starved females consuming the same prey (*P* > 0.05) (Fig. 2).

**Fig. 3:**
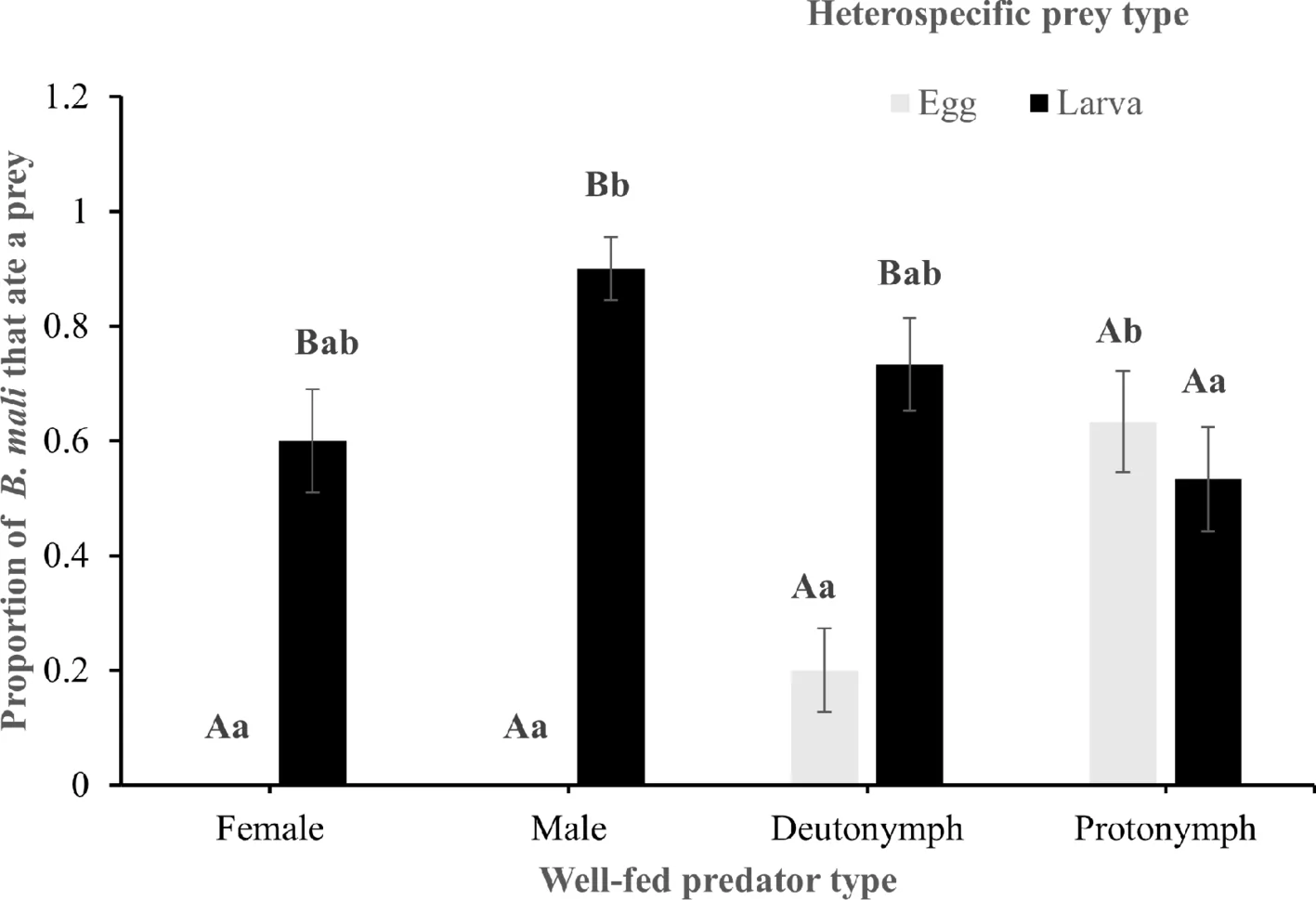
Mean (*± SE*) proportion of *Blattisocius mali* life stages (*n = 30) that ate heterospecific prey. Different uppercase letters indicate significant differences between prey types eaten by each predator, while lowercase letters indicate significant differences among life stages of *B. mali* preying on the same prey (*P* < 0.05; Tukey test)

When the behaviour of various life stages of *B. mali* was tested towards heterospecific prey, the proportion of *B. mali* consuming heterospecific prey was significantly affected by the prey type (*χ²* = 59.2, df = 1, *P* < 0.0001) and life stage of *B. mali* (*χ²* = 13.2, df = 3, *P* < 0.01) as well as their interaction (*χ²* = 52.3, df = 3, *P* < 0.0001) (Fig. 3). Notably, a much higher proportion of *B. mali* protonymphs consumed heterospecific eggs compared to deutonymphs, females, or males (*P* < 0.05) (Fig. 3). Conversely, the proportion of *B. mali* males consuming heterospecific larvae was significantly higher than that of *B. mali* females, deutonymphs, or protonymphs consuming the same prey (*P* < 0.05) (Fig. 3). Furthermore, *B. mali* deutonymphs, females, or males showed a significantly higher tendency to prey on heterospecific larvae than on eggs (*P* < 0.05). In contrast, the proportion of *B. mali* protonymphs that ate heterospecific larvae was similar to that of those consuming heterospecific eggs (*P* > 0.05).

**Fig. 4:**
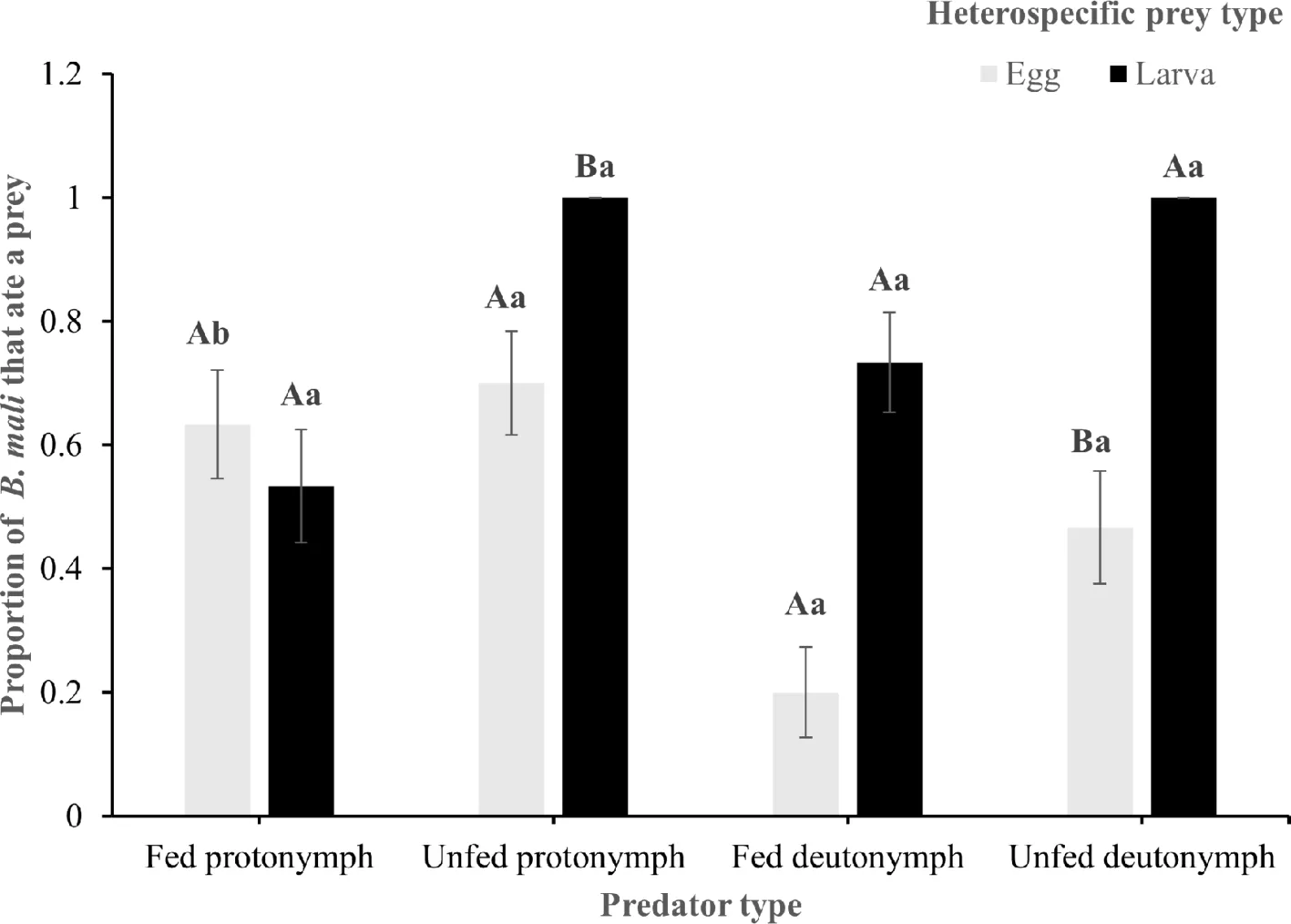
Mean (*± SE*) proportion of fed or unfed protonymphs and deutonymphs of *Blattisocius mali* (*n = 30) that ate heterospecific prey. Different uppercase letters indicate significant differences between fed and unfed protonymphs or deutonymphs of *B. mali* preying on the same prey, while lowercase letters indicate significant differences between fed/unfed protonymphs and deutonymphs of *B. mali* preying on the same prey (*P* < 0.05; Tukey test)

When the behaviour of *B. mali* immatures was evaluated towards heterospecific prey, it was noted that the proportion of *B. mali* immatures that ate heterospecific eggs was significantly influenced by the life stage (*χ²* = 14.0, df = 1, *P* < 0.001). However, neither the starvation period (*χ²* = 3.3, df = 1, *P* = 0.06) nor its interaction with the life stage (*χ²* = 0.02, df = 1, *P* > 0.05) had a significant effect. In contrast, the proportion of *B. mali* immatures that ate heterospecific larvae was significantly affected by starvation period (*χ²* = 35.5, df = 1, *P* < 0.0001). Neither the life stage (*χ²* = 2.6, df = 1, *P* = 0.10) nor its interaction with starvation period (*χ²* = 0.4, df = 1, *P* = 0.99) was significant. The proportion of unfed and fed protonymphs consuming heterospecific eggs did not differ significantly (*P* > 0.05) (Fig. 4). However, unfed protonymphs were significantly more likely to consume heterospecific larvae compared to fed protonymphs (*P* < 0.05). Similarly, unfed deutonymphs had a significantly higher rate of consuming heterospecific eggs than fed deutonymphs (*P* < 0.05), but there was no significant difference in larval consumption between unfed and fed deutonymphs (*P* > 0.05). Moreover, fed protonymphs showed a higher tendency to consume heterospecific eggs than fed deutonymphs (*P* < 0.05) (Fig. 4). Yet, the rates at which fed protonymphs and fed deutonymphs consumed heterospecific larvae did not differ significantly (*P* > 0.05). Finally, the proportions of unfed protonymphs and unfed deutonymphs consuming either heterospecific eggs or larvae were statistically similar (*P* > 0.05).

### Preference of *Blattisocius mali* to conspecific and heterospecific prey

**Fig. 5:**
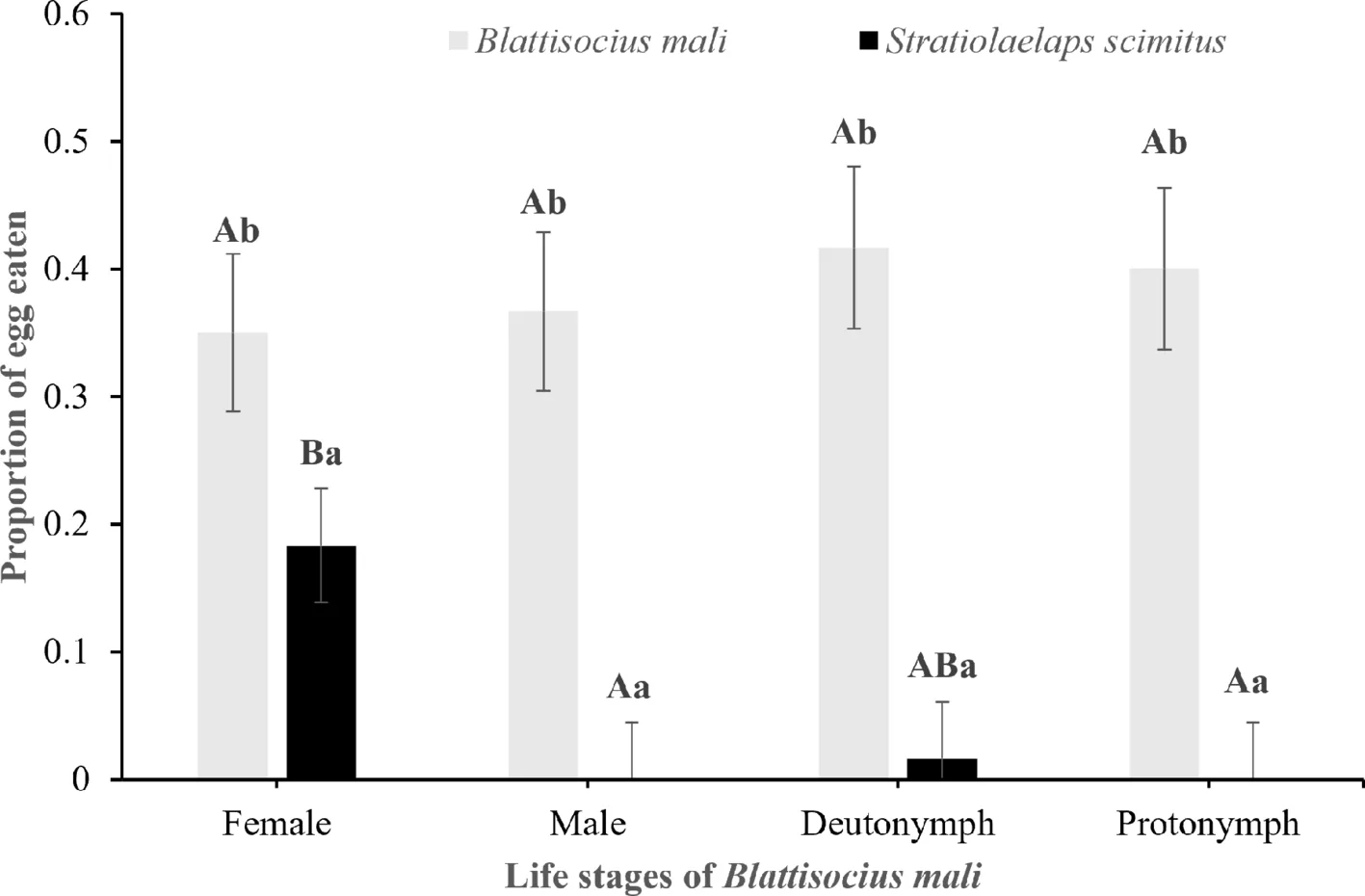
Mean (*± SE*) proportion of conspecific (*Blattisocius mali*) and heterospecific (*Stratiolaelaps scimitus*) eggs eaten by different life stages of *B. mali* (*n = 20) held singly in Plexiglass cages for 24 h. Different uppercase letters indicate significant differences among life stages of *B. mali* that ate the same prey, while lowercase letters indicate significant differences between the proportion of conspecific and heterospecific eggs eaten by *B. mali* (*P* < 0.05; Tukey test)

When examining the preference of various life stages of *B. mali* towards conspecific and heterospecific eggs, it was found that the proportion of eggs eaten was significantly affected by the prey type (*χ²* = 87.5, df = 1, *P* < 0.0001) and its interaction with predator life stage (*χ²* = 25.4, df = 3, *P* < 0.0001). However, the effect of predator life stage (*χ²* = 2.7, df = 3, *P* = 0.44) was nonsignificant. All life stages of *B. mali* ate a significantly higher proportion of conspecific eggs than heterospecific ones (*P* < 0.05). Notably, across life stages, *B. mali* females consumed the highest proportion of heterospecific eggs (Fig. 5).

**Fig. 6:**
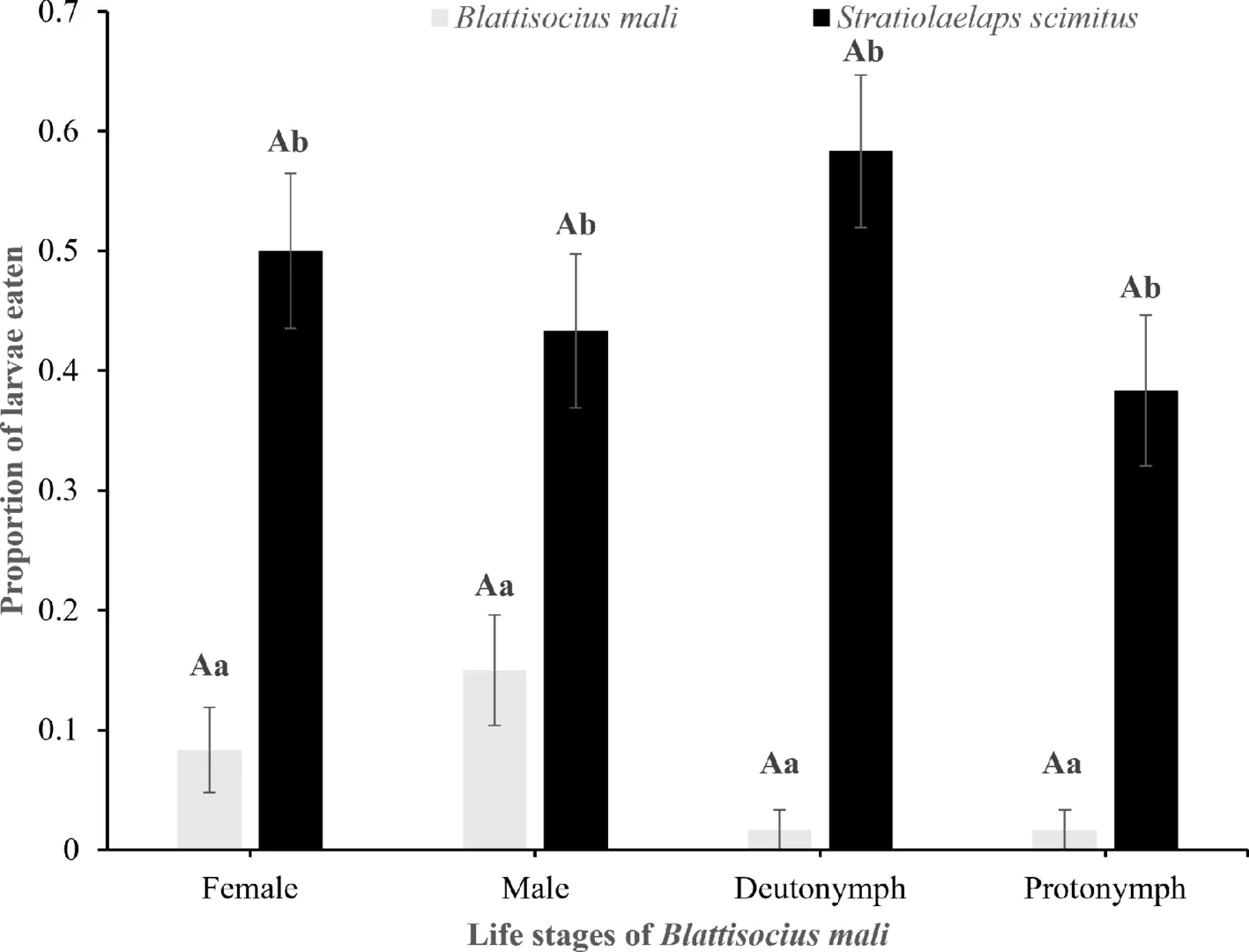
Mean (*± SE*) proportion of conspecific (*Blattisocius mali*) and heterospecific (*Stratiolaelaps scimitus*) larvae eaten by different life stages of *B. mali* (*n = 20) held singly in Plexiglass cages for 12 h. Different uppercase letters indicate significant differences among life stages of *B. mali* that ate the same prey, while lowercase letters indicate significant differences between the proportion of conspecific and heterospecific larvae eaten by *B. mali* (*P* < 0.05; Tukey test).

When assessing the preference of various life stages of *B. mali* towards conspecific and heterospecific larvae, it was observed that the proportion of larvae eaten was significantly affected by the prey type (*χ²* = 112.1, df = 1, *P* < 0.0001) and its interaction with predator life stage (*χ²* = 12.2, df = 3, *P* < 0.01). However, the effect of predator life stage (*χ²* = 4.3, df = 3, *P* = 0.23) was nonsignificant. All life stages of *B. mali* ate a significantly higher proportion of heterospecific larvae than conspecific ones (*P* < 0.05). All life stages of *B. mali* ate a similar proportion of conspecific or heterospecific larvae (*P* > 0.05) (Fig. 6).

## Discussion

The results of the current study revealed that the propensity of *B. mali* females to eat heterospecific eggs was not significantly affected by the starvation period. However, their propensity to eat heterospecific larvae increased with starvation periods up to 48 h, after which it plateaued. Across all starvation periods tested, *B. mali* females, males, or deutonymphs showed a greater propensity to feed on heterospecific larvae than on eggs. Notably, the proportion of *B. mali* males that ate heterospecific eggs was similar to that of females, but when it came to larvae, the proportion of well-fed males was higher than that of females. Furthermore, the proportion of *B. mali* protonymphs consuming heterospecific eggs was significantly higher than that of deutonymphs, females, or males consuming the same prey. Additionally, the proportion of *B. mali* males that ate heterospecific larvae was significantly higher than that of *B. mali* females, deutonymphs, or protonymphs consuming the same prey. All life stages of *B. mali* clearly discriminated between conspecific and heterospecific eggs or larvae. They preferred to prey upon conspecific eggs and heterospecific larvae when given a choice between conspecific and heterospecific eggs or larvae.

Interactions among predator species can significantly influence predator development and population stability. These relationships should be carefully considered when multiple species are introduced into an environment, especially in biological control scenarios where the disappearance of one species or the persistence of others can impact overall effectiveness (Rosenheim et al. 1995; Schausberger and Walzer 2001). Moreover, to safeguard native fauna, it is crucial to thoroughly assess how introduced predators may interact with indigenous species before their release into agricultural settings. Released exotic predators may potentially outcompete native predators, leading to the exclusion or decline of native species (Brodeur and Rosenheim 2000; Gotoh et al. 2014). Understanding these interactions requires studying their effects on population dynamics through controlled laboratory experiments and real-world applications before making decisions about releasing multiple predator species into greenhouses or fields (Walzer and Schausberger 1999). Although intraguild predation by phytoseiid predators on other predatory mites, as well as their predation preference towards conspecific or heterospecific prey, have been extensively studied (Schausberger 1997, 1999; Schausberger and Croft 1999; Walzer and Schausberger 1999; Negloh et al. 2012; Ghasemloo et al. 2016; Döker et al. 2021; Cai et al. 2026; Gong et al. 2026), there is no report on the intraguild predation of a predatory soil mite on another predatory soil mite sharing similar prey and occupying similar habitats, as well as its predation preference for conspecific or heterospecifics.

Our study demonstrated that the starvation period of *B. mali* affected the intensity of intraguild predation across all developmental stages. This finding aligns with the results of Jena et al. (2026c), who observed that prolonged starvation periods led to increased predation rates of *B. mali* on its own immature forms. Due to difficulty in penetrating egg membranes, some predatory mite eggs are less vulnerable to predation than larvae (Schausberger and Croft 2000Meszaros et al. 2007; Farazmand et al. 2015; Jena et al. 2026c). Similar results were obtained in the present study; Across all life stages, a greater proportion of *B. mali* attacked heterospecific larvae than heterospecific eggs. These results may be explained by the active, random search behaviour of *B. mali.* Since eggs are smaller and immobile, the probability of encountering them by chance is lower, leading predators to prey more frequently on the larger, mobile larvae. Ghasemloo et al. (2016) also reported that both *Phytoseiulus persimilis* (Athias-Henriot) and *Typhlodromus bagdasarjani* (Wainstein and Arutunjan) have higher predation rates on their larvae compared to eggs and protonymphs.

Contrary to our initial hypothesis, a similar or greater proportion of *B. mali* males attacked heterospecific prey than that of females did. The heterospecific eggs might have a thick membrane, making them less favourable prey than the larval stage for both *B. mali* females and males. So, both females and males attacked a very small and similar number of heterospecific eggs. Jena et al. (2026c) showed that *B. mali* females did not experience immediate hunger as males did and usually started feeding after being starved for a longer period than males. This difference in hunger levels may explain why a lower proportion of well-fed females attacked heterospecific larvae compared to well-fed males. However, when females were subjected to prolonged starvation (24 h or longer), their proportion attacking heterospecific larvae was the highest, matching that of males.

Intraguild predation typically relates to differences in predator and prey characteristics, such as predator size and developmental stage, and prey size. Smaller, stationary, or less mobile individuals are generally more vulnerable and more likely to be preyed upon than larger, mature, or more active ones (Polis et al. 1989; Rosenheim et al. 1995; Gu et al. 2024). Contrary to the initial hypothesis, this study showed that *B. mali* protonymphs exhibited a higher predation rate on eggs, whereas males showed a greater tendency to prey on larvae. Although *B. mali* protonymphs became hungry more quickly than other life stages, *S. scimitus* larvae (0.30–0.50 mm) were bigger in size than *B. mali* protonymphs (0.25 to 0.35 mm) and thus less prone to predation compared to *S. scimitus* eggs. This size difference might result in a lower predation rate of *B. mali* protonymphs on heterospecific larvae.

Our study supports the hypothesis that *B. mali* could differentiate between conspecific and heterospecific larvae, showing a preference for preying on heterospecific larvae. This behaviour might be linked to the predation type of generalist predators (Schausberger 1999; Walzer and Schausberger 1999). Several generalist phytoseiid mites, including *Typhlodromus athiasae* (Porath and Swirskii), *T. pyri* (Scheuten), *T. swirskii* (Athias-Henriot), *T. phialatus* (Athias Henriot), *T. exhilaratus* (Ragusa), *T. bagdasarjani* (Wainstein & Arutunjan), *Kampimodromus aberrans* (Oudemans), *Euseius scutalis* (Athias Henriot), *Amblyseius degenerans* (Berlese), *N. californicus* (McGregor), and *N. fallacis* (Garman) exhibited discrimination ability showing a clear preference for preying on heterospecifics (Yao and Chant 1989; Schausberger 1997; Schausberger 1999; Walzer and Schausberger 1999; Meszaros et al. 2007; Momen and Abdel Khalek 2009; Ghasemloo et al. 2016). Contrary to our initial expectation, the study showed that *B. mali* preferred to prey on conspecific eggs when given a choice between conspecific and heterospecific eggs. This preference might be influenced by the structural characteristics of the eggs, specifically the thick chorion or the lower nutritional value of heterospecific eggs, which could make these eggs less suitable or more difficult for *B. mali* to prey upon.

In summary, this study shows that *B. mali* can interact with *S. scimitus* and feed on *S. scimitus* eggs and larvae. Additionally, it shows that *B. mali* prefers conspecific eggs over heterospecific eggs and heterospecific larvae over conspecific larvae. Further studies are necessary to investigate the effect of IGP between *B. mali* and *S. scimitus* on their survival, reproduction, population dynamics, and ultimately pest suppression.

## Data Availability

The data used in this study are available by email request to the corresponding author, M.K.J. (jenamanoj401@gmail.com).
